# Roles and perceived responsibilities of investigators and pharmacists in the monitoring of drug-drug interactions in clinical trials - a mixed methods study

**DOI:** 10.1016/j.rcsop.2026.100835

**Published:** 2026-09-12

**Authors:** Anne-Jaël Elskamp, Shirley Cao, Gijsbert Schouten-Ritskes, Joris van der Post, Jacqueline Hugtenburg, Niels Vermeer

**Affiliations:** a Dept. of Pharmacy and Clinical Pharmacology, Amsterdam UMC, De Boelelaan 1118, 1081 HZ Amsterdam, the Netherlands; b Medical Research Ethics Committee (MREC) Amsterdam UMC, Amsterdam UMC, De Boelelaan 1118, 1081 HZ Amsterdam, the Netherlands

**Keywords:** Drug-drug interaction, Investigational medicinal product, Clinical trials, Pharmacists, Clinical investigators

## Abstract

**Purpose:**

To explore the roles and perceived responsibilities of clinical investigators and pharmacists in the monitoring of drug-drug interactions (DDIs) between study medication and concomitant medication in clinical trials. Secondly, sharing of information on study medication treatment with community and hospital outpatient pharmacies was investigated.

**Methods:**

We performed a mixed methods study comprising qualitative semi-structured interviews with clinical investigators and online questionnaires among hospital pharmacists and community and hospital outpatient pharmacists.

**Results:**

Interviews with investigators show they generally consider the current way of DDI monitoring in trials to be adequate. However, they emphasized the need to better define responsibilities and further digitalize monitoring. A total of 43 responses were received from hospital pharmacists (response rate: 62.3% [43/69]). One response was excluded because the hospital did not dispense any study medication. Approximately half of the hospital pharmacists (45.2%, [19/42]) routinely conduct DDI monitoring with concomitant medication when dispensing study medication for patients enrolled in clinical trials. A total of 49 responses were received from community and outpatient pharmacists. Most of the responding pharmacists (44/49 [89.8%]) had been informed about study medication use by a patient in the preceding year. Community and outpatient pharmacists however noted that background information on potential DDIs with study medication is often lacking (19/44 [43.2%]).

**Conclusion:**

Our study highlights critical gaps in the management of DDIs related to the use of study medication with concomitant medication. Clearer allocation of responsibilities, improved digital integration, and sharing of study medication related information may strengthen clinical trial data validity and improve patient safety.

## Highlights

• Management of drug-drug interactions during clinical trials is subpar.
• Improving this strengthens clinical trial data validity and patient safety.
• Clearer responsibilities and improvement of digital integration is necessary.
• Pharmacists should become core member of the clinical trial team.
• In this role they should coordinate DDI evaluation and information sharing.

## 1 Introduction

Clinical trials are essential in the development and evaluation of new and existing pharmacotherapies.^1^, ^2^ In these trials, the effect of one or more experimental or authorized investigational medicinal products is investigated (also: study medication) under strictly regulated conditions as set out in the trial protocol. Patients enrolled in these trials frequently use concomitant medication alongside their study medication. Especially in the case of polypharmacy, this significantly increases the risk of drug-drug interactions (DDIs).^3^, ^4^, ^5^ Undetected and unmonitored DDIs pose a risk for patient safety^6^, ^7^ as this may cause adverse effects or may impact on the efficacy of the treatment^8^, ^9^ and ultimately influence the validity of clinical trial data when the DDI effects are not accounted for in the analysis.

Current literature indicates that DDIs are a significant concern in clinical trials, with studies showing a considerable number of missed interactions.^10^, ^11^ A Dutch single-center study (including 75 participants from 11 clinical trials) found that 45.3% [34/75] of participants had at least one clinically relevant DDI between study medication and concomitant medication. Furthermore, the dispensing community pharmacist was not aware of the use of study medication for 58 out of 75 (77.3%) participants.^11^ In another study performed by the South West Oncology Group in the United States, the incidence of DDIs in two clinical trials was between 18,6% [31/167] and 28,7% [48/167] of patients, depending on the detection method used. A subsequent pharmacist review indicated that 7.2% of subjects [12/167] had a clinically relevant interaction.^10^ In another retrospective study of 128 patients enrolled in 35 US National Clinical Trials Network oncology clinical trials, Lexicomp identified a clinically relevant interaction in 9.4% of patients (12/128), of whom 3.9% (5/128) had an interaction that affected study medication efficacy or toxicity. By contrast, according to the information provided in the study protocol, only 3.1% (4/128) of the patients had a DDI that may have impacted study medication efficacy or toxicity. These findings show that the protocol may not offer sufficient guidance to identify all potential DDIs, which could ultimately influence the validity of trial data and impact patient safety.^12^ It has been shown that the risk of DDIs can be reduced by active participation of a pharmacist by means of medication reconciliation before study enrollment.^13^

However, there is a lack of guidelines on how to organize the medication safety of trial participants in relation to the potential for DDIs between study medication and concomitant medications. It is unknown who is responsible for DDI monitoring and management and it is unclear how this uncertainty translated into clinical practice in various settings. To our knowledge, this study is the first to investigate this. The Good Clinical Practice (GCP) guidelines,^14^ which principles have been adopted in the European Clinical Trial Regulation (ECTR),^15^ considers the investigator responsible for the management of study medication, and ensuring patients are treatment according to the study protocol (which holds information about DDI management). Pharmacist involved in dispensing study medication may however consider it their task to perform DDI monitoring of study medication as required by Dutch law.^16^ Their professional standard requires pharmacists to perform an appropriateness review, including monitoring for potential DDIs, when dispensing medication, regardless of whether this is study or concomitant medication.17., 18. For registered medicines, this is done using the Pharmacy Information System and national databases.19., 20. However, these do not include study medications. DDIs may arise when medication changes are made in participants using both study and concomitant medications, and this is not signaled on time. In addition, the obligation to take every precaution to protect the privacy of trial participants,^15^ may conflict with the necessity to share information with other health care professionals (including community and hospital outpatient pharmacists) preventing adequate information transmission between health care professionals, which could lead to missed DDIs.

Considering the above, it is currently unclear whether and to what extent different stakeholders are responsible for and involved in monitoring of DDI of study medication in clinical practice, and how information regarding study medication use is shared between health care professionals. In the current study we therefore aimed to explore the roles and perceived responsibilities of clinical investigators and pharmacists in the monitoring of DDIs between study medication and concomitant medication in clinical trials. Additionally, sharing of information on study medication use with community and hospital outpatient pharmacists was investigated.

## 2 Methods

This mixed-methods study uses quantitative and qualitative approaches to gather data from the various health care professionals involved in DDI monitoring.

1. Semi-structured interviews were held with clinical investigators at the Amsterdam UMC. Data was collected between December 2024 and Januari 2025.
2. A qualitative questionnaire was distributed among hospital pharmacists from 9 to 29 January 2025.
3. A qualitative questionnaire was distributed among community and hospital outpatient pharmacists from 9 January to 7 May 2025.

### 2.1 Participants and recruitment

Clinical investigators were selected based on their experience in clinical trials, ensuring a mix of different perspectives. Hospital pharmacists were invited individually via the Dutch Association of Hospital Pharmacists (In Dutch: Nederlandse Vereniging voor Ziekenhuisapothekers [NVZA]).^21^ An invitation was sent to a single pharmacist from each of the 69 Dutch hospitals. In the Netherlands, every hospital has one or more pharmacists that are responsible for the clinical trials in their hospital. The NVZA has a member's portal including a list of the pharmacists in the hospital. We aimed to identify a pharmacist responsible for clinical trials in every hospital and sent them an invitation. Invitations to the questionnaire for community and hospital outpatient pharmacists were included in the newsletters that are sent to all the members of the Royal Dutch Pharmacists Association and Academic Networks of pharmacists in the regions of Leiden (LANA), Amsterdam (Saphia), and Groningen (ANNA).

### 2.2 Data collection

Semi-structured interviews were conducted between December 2024 and January 2025 by SC (pharmacy master student), according to an interview template (see online supplementary information). The sample size was determined pragmatically, reflecting the time available from investigators as well as the timeframes of the current study, rather than through a formal sample size or data saturation analysis. The structure of the template was based on the items of the questionnaire and improved with feedback after a mock interview. The interviews were conducted face to face or through Microsoft Teams and recorded using Philips Voice Recorder app. An interview diary was kept for noteworthy observations. Transcription was done using Philips SpeechExec Transcribe software. Recordings of the interview were deleted after intelligent verbatim transcription. Two online, pseudonymized questionnaires were developed for hospital pharmacists and community/hospital outpatient pharmacists, respectively, using Castor EDC (v2024.4.1.1). Data collection for hospital pharmacists lasted from 9 to 29 January 2025. Data collection for community and hospital outpatient pharmacists lasted from January to May 2025. The questionnaire collected data on demographics, DDI screening practices and information sharing in relation to the use of clinical study medication.

### 2.3 Data analysis

Interview transcripts were analyzed independently by two researchers (SC and GSG) using MAXQDA 24 (Release 24.5.1). The analysis approach used followed reflexive thematic analysis (TA) as described by Braun and Clarke.^22^ An inductive approach was used, which provided an open-coded analysis. The design of the interview template did lead to some deductive analysis, as the template was made to match the questionnaires for the community and hospital outpatient pharmacists, aiming for themes associated with the research objective.^23^

All questionnaires completed ≥75% (hospital pharmacists) and ≥ 50% (community/hospital outpatient pharmacists) were included in the analysis. These thresholds reflect the percentage of completed questions answered by each participant. Different completion thresholds were applied pragmatically because the two questionnaires differed in structure as the hospital pharmacist version contained fewer open-ended items and thus had inherently higher completion rates. Applying a uniform threshold would exclude a disproportionate number of community/outpatient pharmacist responses. The chosen thresholds are intended to exclude respondents who opened but did not meaningfully complete the questionnaire, while retaining an adequate sample size in both groups. Descriptive analysis methods were used to analyze the qualitative data collected from the questionnaires. IBM SPSS® Statistics software was used to calculate frequency, percent, mean, standard deviation, minimum, maximum. Quantitative data from these questionnaires consist of open-ended questions or additional open remarks. These remarks and answers were analyzed using MAXQDA, following the same reflexive thematic analysis as performed with the interview transcripts. The reflexive thematic analysis was used to find underlying patterns in all answers.

### 2.4 Ethics approval

The study protocol was approved by the Medical Review Ethics Committee (MREC) of the Amsterdam University Medical Centre (Amsterdam UMC) (application number 2024.1099). This study did not fall within the scope of the Medical Research Act with People (in Dutch: Wet Medisch-wetenschappelijk Onderzoek met mensen, WMO), so non-WMO approval was obtained.

## 3 Results

### 3.1 Clinical investigators

**Fig. 1:**
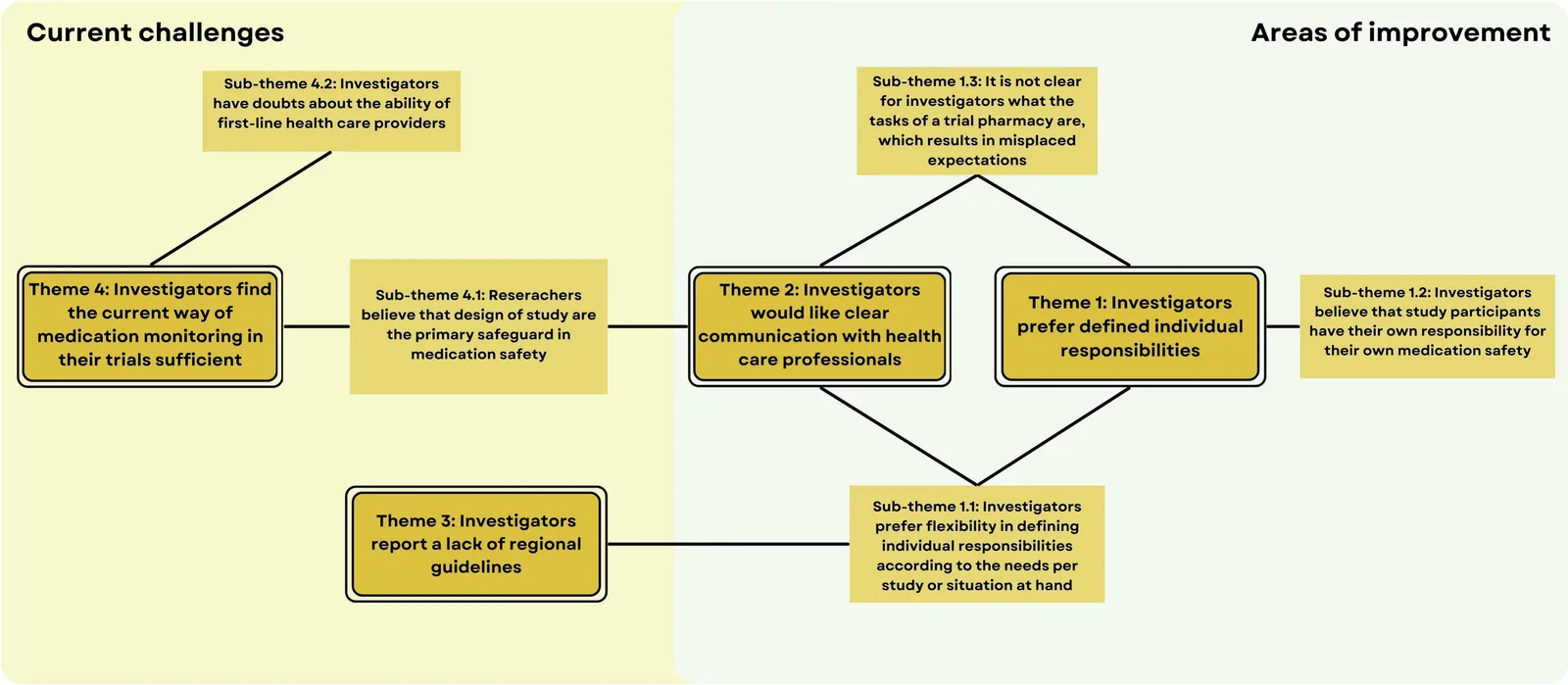
Visualization of themes and sub-themes observed in the interviews with clinical investigators.

Interviews were conducted with five Amsterdam UMC clinical investigators from different clinical departments. The interviews lasted between 22 and 35 min (average: 26.2 min). A total of four themes and seven sub-themes are identified as seen in Fig. 1. The four main themes were (1) the investigators prefer defined individual responsibilities, (2) investigators would like clear communication with other health care professionals, (3) investigators report a lack of regional guidelines and (4) investigators find the current way of medication monitoring in their trial sufficient. All themes and their subthemes are explained in the sections below.

#### 3.1.1 Theme 1: Individual responsibilities of stakeholders

This theme highlights that clinical investigators believe that every stakeholder is important in making clinical trials run as smoothly and safely as possible, but to make them run as clearly and efficiently as possible, their individual responsibilities should be more clearly defined. There are three subthemes connected to this theme. The requirement of flexibility, the responsibility of trial participants and the lack of knowledge on the tasks of the pharmacy.

##### 3.1.1.1 Sub-theme 1.1: Requirement of flexibility in defining individual responsibilities of health care professionals according to the needs per trial or situation at hand

Clinical trials vary in design, risk level, and trial population, therefore each trial needs different services and different involvement of health care professionals. Thus, even though clinical investigators prefer clearly defined individual responsibilities, these should also be flexible and adaptable to the specifics of each trial and the situation at hand.

“I do think you need to be flexible in that regard, and it is not really a ‘one-size-fits-all’ approach, but it could be a more explicit part of a study protocol, for example. So, you think in advance about how medication safety will be ensured, including interactions, kidney, and liver function issues, the CYP profiles, etc., and how that will be guaranteed. That you think about it and make a plan for each study; I think that is definitely possible.”

– Interviewee

##### 3.1.1.2 Sub-theme 1.2: Trial participants are also responsible for their medication safety

Clinical investigators expressed that it is important to take the responsibility to make the use of study medication as safely as possible, but that a lot of responsibility still comes down to the trial participant. Patients are informed about the risks of participating in a clinical trial and the use of study medication both verbally and by means of the Patient Information File (PIF) and are therefore informed about their responsibilities.

“On the other hand, the participant also has responsibility. So, that might be complicated. Just like you cannot stand next to a participant to see if they are truly taking it [the study medication] as they are supposed to. You give advice about it, but you can't control everything.”

– Interviewee

##### 3.1.1.3 Sub-theme 1.3: The tasks of the trial pharmacy are unclear, which results in misplaced expectations

Clinical investigators stated several times that ‘the pharmacy will take care of that’, only to find out that that was not the case. It is often unclear for clinical investigators what the tasks of a trial pharmacy are. They are unaware of the challenges the trial pharmacy faces and the tasks that are (im)possible for the pharmacy to perform. This results in the misplaced expectation that the (hospital) pharmacy will take responsibility for nearly all aspects of study medication use.

“Yeah, interactions. I assume they [trial pharmacy] might, I do not know if they look at that, usually they look at everything. So, they are probably doing that as well, but I wouldn't know.”

– Interviewee

#### 3.1.2 Theme 2: Communication with other health care professionals

The second theme focusses on communication. Clinical investigators stated that clear communication with colleagues in the research context is vital for the monitoring of medication safety, as each health care professional has their distinct expertise.

“But the same applies here, you need to keep each other well-informed and track what medications are being prescribed.”

– Interviewee

##### 3.1.2.1 Sub-theme 2.1: The need for better electronic communication tools to facilitate the sharing of trial information

When brought to their attention, several clinical investigators felt that a lot of challenges could be resolved by making information more readily available to other health care professionals. One of the proposals is a national EPD, in which every health care professional has access to the same essential information. Others deemed the current electronic prescription systems (i.e., hospital information systems) not suitable for the current demands of information sharing and advocated for improvements of these systems and other electronic communication tools.

“But indeed, I think the communication between the general practitioner, hospital pharmacy, community pharmacy, doctor, research nurse… the only thing that could help is a nationally shared patient file. We are working on that again, after it was once rejected by the minister, but I do not know how far it is progressed.”

– Interviewee

#### 3.1.3 Theme 3: A lack of regional guidelines

All clinical investigators were unaware of any regional guidelines. Two clinical investigators were worried they might have missed a guideline and expressed that a regional guideline would be beneficial.

#### 3.1.4 Theme 4: The current way of monitoring the use of study medication is adequate

Clinical investigators believe that the medication safety in their trials is safeguarded by implementing various safety assuring measures and adhering to agreements made prior to the trial. However, until it was brought to their attention by the interviewer, they had not been aware of the possible shortcomings in the current way of monitoring study medication safety, based on existing literature. Two subthemes can be connected to this theme: the primary safeguard of the trial design and the role of the general practitioner (GP).

##### 3.1.4.1 Sub-theme 4.1: An adequate trial design is the primary safeguard in medication monitoring

Clinical investigators stated four factors that guarantee the safety of study medication use in their trials. First, the in- and exclusion criteria are designed to eliminate potentially harmful DDIs. Second, four of the clinical investigators mentioned that in most cases the study medication used in their trials does not cause many DDIs. Third, trial designs usually make for frequent patient contacts, notably in phase I and II trials. As patients are seen up to two times a week, clinical investigators have ample opportunity to monitor the health status of the participants. This works particularly well in combination with the fourth factor: the agreements that are made with trial participants. They are instructed to always inform the clinical investigator of any change in medication or in health status. However, a downside to this system is that its success depends on the honesty and alertness of individual participants. Clinical investigators also admitted that this system is not foolproof, especially in phase III and IV trials, and when in later stages patient contacts become less frequent.

“At each visit, the presence of new medication is checked. However, it is also possible that a patient has already received new medication prior to the visit, and they may mention it. This could create a situation where an interaction may occur. Therefore, relying solely on the clinical investigator to inquire about medication use does not completely eliminate the risk. Between visits, a general practitioner or another medical specialist may initiate a new medication […], which could lead to a potential interaction.”

– Interviewee

##### 3.1.4.2 Sub-theme 4.2: The capacity and willingness of first-line health care professionals to read and understand study medication information

Clinical investigators communicate trial information to the GP when a patient participates in a clinical trial, specifically at the start and at the end of the trial using electronic messaging. Several clinical investigators expressed uncertainty about whether GPs fully read their messages, with one noting that GPs often lack the time to do so. The second point raised by clinical investigators was that study medication is often more complex and uncommon than medication used in the first line. As a result, they do not expect health care professionals in primary care to be able to familiarize themselves with this medication in addition to their other responsibilities. Furthermore, it is often not possible to have access to information about study medication and its use, as it pertains to sensitive and confidential data. None of the clinical investigators communicate trial information to the community pharmacists.

“GPs, in fact, do not want to know that much, as they receive hundreds of such messages per month from various patients, and they do not read the entire message, as has been shown. […] The GP primarily reads the first part of the letter and does not go through my entire epistle, where I explain the specific type of illness, why the patient is in this study, what the study medication aims to do, and the likelihood of response.”

– Interviewee

### 3.2 Hospital pharmacists

A total of 43 responses were received from hospital pharmacists (response rate: 62.3% [43/69]). One response was excluded because the hospital did not dispense any study medication. Responses were received from 11 out of the 12 provinces in the Netherlands. Of the responses, 18 were from general hospitals, 17 were from teaching hospitals and seven from academic hospitals. An average of 77 clinical trials were conducted per hospital [range: 1–400]. Hospital pharmacists handled DDI checking in various ways (Table 1). Overall, less than half (19/42, 45.2%) of hospital pharmacists were involved in interaction checking in any stage of the use of study medication.

**Table 1:** Involvement of hospital pharmacists in DDI monitoring of study medication and DDI information sharing.

Involved in interaction checking any stage	19/42 (45.2%) *
Enter study medication in the hospital information system	16/19 (84.2%)
Using a trial specific manual for interaction checking	5/19 (26.3%)
Using the study protocol	4/19 (21.1%)
Using an online interaction checker	2/19 (10.5%)

Involved in information sharing at any stage	16/41 (39.0%) **
Hospital pharmacy sends information using a national medicine database	9/16 (56.3%)
Hospital pharmacy sends a message to the community or hospital outpatient pharmacist	5/16 (31.3%)
Information sharing is done via the hospital outpatient pharmacist	3/16 (18.8%)
Hospital pharmacy hands the patient a letter to the GP	3/16 (18.8%)
Other***	2/16 (12.5%)

*Percentages do not add up to 100% since these are multiple response questions. One respondent did not specify the method of interaction checking. ** one missing value. *** other includes instructing the patient to inform the community pharmacy (*n* = 1) and sending a message to the GP (*n* = 1). Abbreviations: DDI = drug drug interaction, GP = general practitioner.

Among the 22 hospital pharmacists not involved in DDI monitoring, this was most frequently (17/22, 77.3%) because DDI monitoring by the clinical investigator was considered adequate. Other reasons were that monitoring was already included in the protocol (10/22, 45.5%), that study medication could not be entered into the hospital information system (10/22, 45.5%), and that it was impossible to perform the monitoring because a complete medication record was not available (8/22, 36.4%). A poignant nuance given by two respondents in the free text option was that there is a difference in how the medication of outpatients and hospitalized patients can be monitored, as the medication of outpatients cannot be viewed in the hospital information system. If DDI monitoring is performed, the majority of the respondents use the hospital information system. Creative ways need to be used to make monitoring with the hospital information system possible. For example, by substituting study medication for (registered) non-study medication for which DDI checking is available which allows for electronic DDI monitoring of study medication which route, however, also has its limitations.

**Fig. 2:**
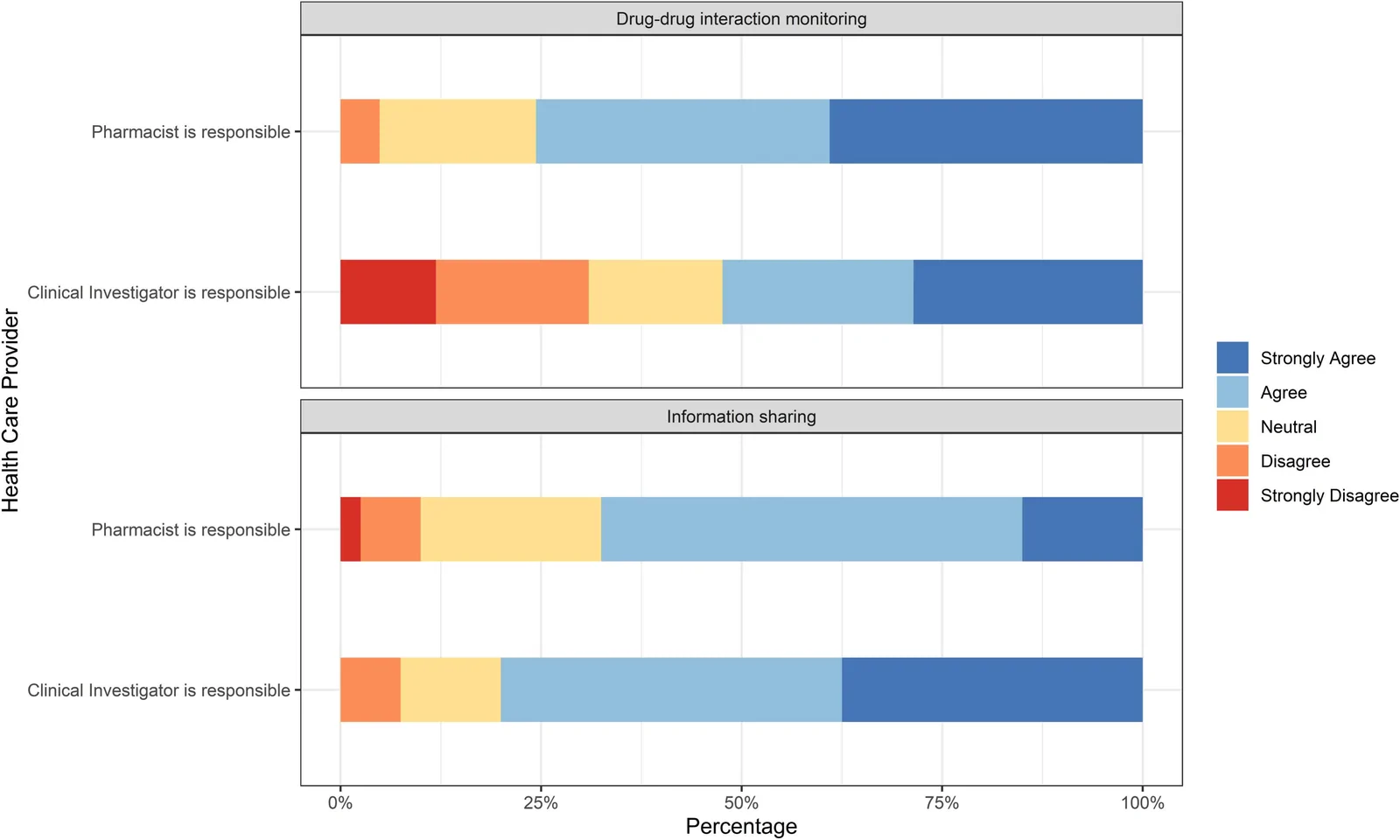
Perceived responsibilities by hospital pharmacists when asked who is responsible for performing DDI monitoring and who is responsible for the sharing of information about study medication to a first line pharmacy.

Overall, 39.0% (16/41, 1 missing value) of respondents were involved in the sharing of study medication information to a participants' community pharmacist and hospital outpatient pharmacist. The most frequent reason given as to why information sharing was not facilitated by the hospital pharmacy was that this information could only be shared by the clinical investigator (11/25, 44.0%). Another reason was that the participants themselves had to provide the clinical investigator with information of their study medication regarding its use and effects elicited (7/25, 28.0%). Several hospital pharmacists (20.0%, 5/25) added that information sharing often is not possible because the (unregistered) study medication cannot be entered into the hospital information system. Most of the hospital pharmacists agreed that the hospital pharmacists should be responsible for DDI monitoring of study medication (see Fig. 2). However, it was generally believed that hospital pharmacists and clinical investigators should share this task. Improvements of the electronic infrastructure, such as the possibility to register trial medicines in the hospital information system and national medicine database, were commonly cited as a major step towards the improvement of medication safety in clinical trials.

### 3.3 Community and hospital outpatient pharmacies

We received 49 responses, most responses from community pharmacists (38, 77.6%) and 10 from hospital outpatient pharmacists (20.4%). One response (2%) was obtained from a participant who selection ‘other institution’, however, as the free-text follow-up question intended to specify this response was left blank, the nature of this institution could not be determined. The response rate was not homogenous across the different regions of the Netherlands: a higher number of responses came from the more populous regions of North- and South Holland. Demographic characteristics can be found in Appendix 1. Most respondents (44/49, 89.8%) indicated to have been informed at least once a year about a patient's use of study medication. Mostly (23/49, 46.9%), this occurred once or twice per year. The pharmacies that indicated to have been informed more than 10 times per year were mainly hospital outpatient pharmacists (5/6, 83.3%). Reversely, only 13.0% (3/23) of pharmacies that were informed once or twice per year were hospital outpatient pharmacists.

When study medication information is received, it may include its name (37/44, 84.1%), contact information of the clinical investigator (32/44, 72.7%), the dosage (26/44, 59.1%), and the period of use (25/44, 56.8%). Information on potential DDIs is often lacking (19/44, 43.2%). In nine pharmacies (9/49, 18.4%) there were instructions on study medication written in their quality manuals. They concerned the performing of checks on DDI (8/9 times, 88.9%) and recording the use of study medication in the pharmacy information system (8/9 times, 88.9%). How to share information on the use of study medication, e.g., an interaction with concomitant medication, was not included as often (2/9 times, 22.2%).

Most of the respondents felt that they lacked essential information on the use of study medication to fulfill their responsibility as a pharmacist to provide adequate pharmacotherapeutic care (26/42, 61.9%, 7 missing values). community and hospital outpatient pharmacists were also asked how DDI monitoring and information sharing with regard to study medication should be done and which challenges they expected to encounter. Their responses can be divided into two themes, with two subthemes under theme 1. The first theme exposes the uncertainty on who is responsible for the study medication-related DDIs of these patients and the second theme shows that community and hospital outpatient pharmacists do try to perform DDI monitoring.

#### 3.3.1 Theme 1: Uncertainty about responsibility to monitor study medication-related DDI and information sharing of study medication data

Some community and hospital outpatient pharmacists felt a responsibility (mostly with regard to keeping the patient's medication record up to date), whereas others thought the responsibility should be with the clinical investigators.

“In my view, the greatest responsibility for monitoring study medication lies with the research team. I see it as our role to provide them with information on issues with the study medication that are identified in the pharmacy. Indeed, we can monitor whether a patient's medication that may interact with the study medication, is not dispensed. Unwanted medications should then of course have been communicated to us in advance.”

“The community pharmacy is the place that has a complete overview of all concomitant medication, so a short line [of communication] to the trial site is necessary for the sharing of information and proper monitoring of both the concomitant and study medication used, both by the trial site and the community pharmacy.”

This theme can be connected to two subthemes, where the first one highlights a gap in the current system as experiences by these pharmacists and the second one points to a possible solution to bridge this gap.

##### 3.3.1.1 Subtheme 1.1: adequate information to monitor study medication use and DDIs is lacking

Community and hospital outpatient pharmacists, community pharmacists in particular, observed many challenges when asked about information sharing and the monitoring of study medication use and DDI's related to study medication. This is mainly because they believe that to perform these tasks they should be adequately informed about the study medication and its use (duration, dosage, name, interactions, etc.).

“If sufficient information on study medication will be made available, it can be added to the medication monitoring of medication dispensed by the pharmacy. However, this information is not currently available. At the moment, no possibility to implement full medication monitoring.”

##### 3.3.1.2 Subtheme 1.2: study medication should be listed in the national medicine database, facilitating easy information exchange between health care professionals

If community and hospital outpatient pharmacists are required to participate in DDI monitoring and share information about study medication-related DDIs, it should be performed in the current (electronic) way of monitoring non-study medication. This ties into the first subtheme, that community and hospital outpatient pharmacists prefer to receive information on the use of study medication by the same channel as used for information on the use of concomitant medication: i.e. by means of the National Exchange Point (in Dutch: Landelijk Schakelpunt Patiëntgegevens, LSP), as this national portal is considered the most appropriate way to receive updates on the use of study medication. This leads to the second subtheme: new, unregistered medicines used in trials should also be included in the national medicine database, because only then is it possible to share information by using the LSP. In this case the LSP can be used as a source of information on medication used, including pharmacovigilance data.^24^

“The processing of registered study medication is clearer/easier than unregistered study medication. If study medication is not registered, it is not possible for us to process it correctly.”

This issue also arose when community and hospital outpatient pharmacists indicated that it would be it more difficult to perform DDI monitoring for unregistered study medication than for registered study medication, because the data and resources required for the electronic screening of interactions caused by the former medication are not available in the pharmacy information systems.

“Unregistered study medication cannot be entered into the pharmacy information system as ‘active medication’, which means that DDI screening cannot be performed using the pharmacy information system.”

#### 3.3.2 Theme 2: community and HOPs try to perform DDI monitoring in numerous ways, but none are adequate

The last theme describes how despite the various challenges (see above), community and hospital outpatient pharmacists often manage to perform DDI monitoring of study medication through creative ways. Examples of these include the entering of study medication and data on potential interactions manually and making memos in patient files. However, these ways vary per individual community pharmacist and hospital outpatient pharmacist as there are no guidelines or protocols.

## 4 Discussion

Checking for possible DDI when dispensing medicines contributes significantly to the safe use of medication. The present study, however, highlights the existence of critical gaps in the management of DDI related to the use of study medication concurrently with concomitant medication, particularly regarding the responsibilities of clinical investigators and the various pharmacists involved. Although pharmacists should play a vital role in DDI monitoring, in clinical trials their involvement varies, with some hospital pharmacists shifting responsibilities to clinical investigators. Furthermore, because often only GPs are informed about trial participation, community and hospital outpatient pharmacists are often not aware that their patients participate in a clinical trial and use study medication. Moreover, information on study medication, notably if it is not registered, is not readily available for electronic DDI monitoring. The lack of a standardized approach for communicating study medication data between health care professionals as well as the limited involvement of pharmacists in clinical trial teams further complicates the issue.

These findings are consistent with those of previous studies reporting significant health risks in clinical trials due to missed DDIs.^11^, ^12^ The unavailability of information on unregistered study medication in the national medicine database and consequently, the lacking possibility of accessing it using the hospital or pharmacy information system, and inability to register this study medication use in these systems, is a significant barrier to effective DDI monitoring. Clinical investigators' reliance on study protocols and patient self-reporting also contributes to the lack of comprehensive DDI monitoring, particularly in later stages of phase III and IV trials when patient visits become less frequent. The study protocols are often incomplete which entail a high risk of insufficient medication safety handling.^25^ All participants (clinical investigators, hospital pharmacists, community, and hospital outpatient pharmacists) expressed the need for improvement of digitally available tools, such as the listing of study medication in the national medicine database. This would streamline the process of achieving optimal medication safety, allowing for more efficient communication between health care professionals and better DDI monitoring. In addition, clearer but flexible definitions of responsibilities of the health care professionals involved could improve the coordination of care whereas greater involvement of pharmacists in clinical research teams could reduce the risk of missed DDIs and improve the quality of research outcomes.^26^ A clear benefit of medication reconciliation and pharmacist consultations have also been demonstrated to increase participant safety.^13^, ^27^ In regular care, using the pharmacy information system and data compiled in the ‘G-Standaard’, monitoring DDIs is now highly automated (as listed in the *Z*-Index). In a fully integrated fashion the ‘G-Standaard’ database supports the prescribing, dispensing, ordering, declaring and reimbursement of medicines and other healthcare products in the Netherlands.^19^ Hence, this national medicine database is the core of the various information systems used in the dispensing of medicines to patients in hospitals and elsewhere. In the Netherlands patient medication data are recorded in the pharmacy information system of the pharmacy or pharmacies (see below) where patients are registered. Other health care professionals may view this data (as part of the electronic patient dossier (EPD)) via the LSP if the patient has given informed consent.^20^ At present, the integration of study treatments in this database is not possible.

Based on the findings of the study, we propose a number of recommendations to improve patient safety in clinical trials. The current shortcomings in DDI monitoring of study medication are, in many cases, relatively easy to address, often by formalizing measures that are already in place. Guided by the themes identified in our analysis, these recommendations fall into two categories: the availability of information and the structure of trial teams. Regarding information availability, three improvements have been proposed. First, upon inclusion the use of concomitant medication of each participant, obtained by accessing the medication record via the LSP or by requesting the participant's community pharmacist and/or hospital outpatient pharmacist to provide a medication overview, should be jointly reviewed by the clinical investigators and the hospital pharmacist dispensing the study medication. Second, LSP consent should be part of the clinical trial consent. Third, in addition to the GP, clinical investigators should inform a participant's community pharmacist and/or hospital outpatient pharmacist about their client's participation in a clinical trial and the intended use of study medication. Regarding the structure of trial teams, two further improvements are proposed. First, having extensive knowledge of the study medication, the hospital pharmacist dispensing this medication becomes a standing member of the clinical investigator team conducting the trial and coordinate DDI evaluation and information sharing.^28^ Second, during the trial community and hospital outpatient pharmacists should report relevant changes in the concomitant medication of a trial participant to the hospital pharmacist dispensing the study medication. The hospital pharmacist evaluates the report with the clinical investigator to see if there are possible consequences for the use of the study medication. This also applies to potentially DDI-related adverse events reported to clinical investigators by trial participants. In the case of a relevant DDI, notably if unregistered study medication is involved, the evaluation result and a user recommendation is reported back or communicated to the community pharmacist/hospital outpatient pharmacist and/or hospital pharmacist for implementation. Finally, regarding (electronic) DDI monitoring and the sharing of (trial) medication safety data, it is of utmost importance that the national medicine database is supplemented with the nominal (relevant key data only) listings of unregistered medicines used in clinical trials. Only this allows the electronic recording of various data related to the use of all study medication in the hospital information system/pharmacy information system and the sharing of study medication safety data between health care professionals. The national trial register can be used to compile the nominal listings. This way, all health care professionals involved in the monitoring of study medication safety can, based on their own expertise and a reliable means of communication, fulfill their obligation to adequately contribute to the monitoring of DDI related to the use of clinical study medication.

To our knowledge this study is the first to investigate the roles and responsibilities of several health care professionals regarding DDI management of study medication. This study has several limitations. The focus of this study was on the attitudes and involvement of several stakeholders: clinical investigators affiliated with the Amsterdam UMC, hospital pharmacists, community, and hospital outpatient pharmacists, but the perspectives of trial participants and sponsors are missing. Only a limited number of clinical investigators of the Amsterdam UMC were interviewed, which might provide a limited or skewed view. It is not certain that information saturation has been achieved. However, a diverse range of clinical investigators have been approached, and various study designs were discussed during interviews. Since only clinical investigators at the Amsterdam UMC were included, the results are not representative of other hospitals and other countries. Expanding to a multicenter study would increase the robustness of the results and more widely applicable recommendations. Selection bias may also have occurred, as community and hospital outpatient pharmacists routinely involved in management of DDIs with study medication are more likely to respond to a questionnaire regarding this topic than those who are not. Our finding that almost 90% of the responding community pharmacists/hospital outpatient pharmacists had been confronted with study medication in the previous year likely confirms this. Nevertheless, our findings are highly relevant to most pharmacies, due to the large number of clinical trials conducted in the Netherlands annually. This could lead to two consequences, namely overestimating the number of pharmacies that come across study medication on the one hand, and also overestimating the number of pharmacies that have procedures in place or are involved in DDI checking or information transmission of study medications. Lastly, our study solely focused on DDI management in clinical trials, whereas appropriateness review of study medication should also include the dosage and interval. Although not a subject in our current study, these are also important topics to consider in future guidelines. Future research should build on the current findings. Additionally, a discussion of recommendations using the DELPHI method would strengthen the recommendations.

In conclusion, our study highlights critical gaps in the management of DDIs related to the use of study medication with concomitant medication. These shortcomings can be addressed by implementing a number of simple procedural measures and completing the national medicine database with a nominal list of unregistered trial medication. Additionally, we advocate formulating best-practice guidelines for management of DDI in clinical trials and sharing of information of study medication between health care professionals. These guidelines should include the allocation of responsibilities, improve digital integration, and share relevant information of study medication use with the appropriate health care professionals, where the role of the pharmacists should be more utilized. Ultimately, this will strengthen clinical trial data validity and improve patient safety.

## CRediT authorship contribution statement

**Anne-Jaël Elskamp:** Writing – review & editing, Writing – original draft, Visualization, Supervision, Project administration, Formal analysis, Conceptualization. **Shirley Cao:** Writing – original draft, Visualization, Formal analysis. **Gijsbert Schouten-Ritskes:** Writing – review & editing, Investigation, Formal analysis. **Joris van der Post:** Writing – review & editing, Conceptualization. **Jacqueline Hugtenburg:** Writing – review & editing, Resources, Methodology. **Niels Vermeer:** Writing – review & editing, Supervision, Conceptualization.

## Compliance with ethical standards

The study was conducted in accordance with the Declaration of Helsinki. Approval was granted by the Non-WMO (Medical Research Involving Human Subjects Act, in Dutch: Wet Medisch-wetenschappelijk Onderzoek met mensen) Review Committee of Amsterdam UMC under number 2024.1099. Clinical trial number: not applicable. Informed consent was obtained from all individual participants included in the study for participation in the study and publication of the data.

## Declaration of competing interest

The authors declare that they have no known competing financial interests or personal relationships that could influence the work reported in this paper.

## Appendix A Supplementary data

Supplementary data consists of demographic characteristics of respondents, eCRFs and interview schemes.
