# Supplementary material for: Roles and perceived responsibilities of investigators and pharmacists in the monitoring of drug-drug interactions in clinical trials - a mixed methods study
Source: Explor Res Clin Soc Pharm. 2026 Sep 12;24:100835. doi: 10.1016/j.rcsop.2026.100835 (PMC13602037; doi:10.1016/j.rcsop.2026.100835)
Supplement: Supplementary file 1 [file mmc1.pdf]

SUPPLEMENTARY INFORMATION

SUPPLEMENT 1: Demographic characteristics

Demographic characteristics of the respondents for the questionnaire for community and outpatient pharmacies.

Variable	Options	Count (%)
**Type of pharmacy**		
	Community pharmacy	38 (77.6%)
	Outpatient pharmacy	7 (20.4%)
	Other	1 (2%)
**Geographical distribution (Province of the Netherlands)**		
	North Holland	19 (38.8%)
	South Holland	14 (28.6%)
	Gelderland	6 (12.2%)
	Groningen	3 (6.1%)
	Flevoland	3 (6.1%)
	Other	4 (8.1%
**Type of pharmacy system**		
	Pharmacom	28 (57.1%)
	CGM Apotheek (MIRA)	13 (26.5%)
	Other	8 (16.3%)
**Amount of FTE pharmacist**		
	Less than 2	20 (40.8%)
	2-3.9	12 (24.5%)
	4 or more	8 (16.3%)
	Missing value	9 (18.4%)
**Number of prescriptions per month**		
	<10.000	22 (44.9%)
	10.000-20.000	16 (32.7%)
	20.000-30.000	3 (6.1%)
	30.000-50.000	4 (8.2%)
	Other	4 (8.2%)

SUPPLEMENT 2: Empty eCRF – Community Pharmacy

Survey 'Enquête medicatiebewaking en -overdracht van studiemedicatie - Openbare/poliklinische apotheek en apotheekhoudende huisarts'

Enquête medicatiebewaking en -overdracht van studiemedicatie - Openbare/poliklinische apotheek en apotheekhoudende huisarts - Introduction

---

Welkom bij de enquête van het onderzoek ‘Medicatiebewaking en -overdracht van studiemedicatie’. Het doel van dit onderzoek is het in kaart brengen van hoe de huidige medicatiebewaking en -overdracht van studiemedicatie op dit moment in Nederland is ingericht, met speciale aandacht voor hoe de openbare/poliklinische apotheek en apotheekhoudende huisarts deze processen heeft ingericht. Dit is met het voornemen om aanbevelingen te kunnen doen voor een mogelijke nieuwe richtlijn omtrent studiemedicatie.

- Het zal ongeveer 5-10 minuten duren om de vragenlijst in te vullen.
- De antwoorden worden gecodeerd verzameld en zijn niet herleidbaar naar u of de organisatie waar u werkzaam bent.
- Er wordt zorgvuldig omgegaan met de verzamelde gegevens, conform de Gedragscode Gezondheidsonderzoek (https://www.coreon.org/wp-content/uploads/2022/01/Gedragscode-Gezondheidsonderzoek-2022.pdf).

Let op! Deze vragenlijst graag maar 1 keer per apotheek invullen!

Dit onderzoek wordt uitgevoerd in opdracht van het Amsterdam UMC, afdeling Klinisch Geneesmiddelonderzoek. Bij vragen kan er contact opgenomen met de uitvoerende onderzoeker via e-mail: a.j.elskamp@amsterdamumc.nl.

Enquête medicatiebewaking en -overdracht van studiemedicatie - Openbare/poliklinische apotheek en apotheekhoudende huisarts - Toestemming vragen

Number	Question	Answers
1.1	Hierbij geef ik toestemming voor het gebruik van de ingevulde gegevens voor onderzoek en ben ik me bewust dat de ingevulde gegevens gecodeerd worden verzameld en niet herleidbaar zijn naar mij of de organisatie waar ik werkzaam ben. Er zal zorgvuldig met de verzamelde gegevens omgegaan worden. Door ‘Ik geef toestemming’ te klikken, neemt u deel aan de enquête<br>Indien geen akkoord, mag de enquête afgesloten worden.	◯ Ik geef toestemming

Enquête medicatiebewaking en -overdracht van studiemedicatie - Openbare/poliklinische apotheek en apotheekhoudende huisarts - Algemene vragen

Number	Question	Answers
	Eerst volgen er 6 algemene vragen.	
2.1	Onder welke categorie valt uw apotheek?	◯ Een openbare apotheek<br>◯ Een poliklinische apotheek<br>◯ Een apotheekhoudende huisarts<br>◯ Iets anders
2.1.1	***If 'Onder welke categorie valt uw apotheek?' is equal to 'Iets anders' answer this question:***<br>U heeft bij de vorige vraag 'Iets anders' ingevuld. Licht dit toe.	
2.2	In welke provincie is de apotheek waarin u werkzaam bent gevestigd?	◯ Drenthe<br>◯ Flevoland<br>◯ Friesland<br>◯ Gelderland<br>◯ Groningen<br>◯ Limburg<br>◯ Noord-Brabant<br>◯ Noord-Holland<br>◯ Overijssel<br>◯ Utrecht<br>◯ Zeeland<br>◯ Zuid-Holland
2.3	Welk apotheekinformatiesysteem (AIS) wordt er in uw apotheek gebruikt?	◯ Pharmacom<br>◯ CGM Apotheek (Mira)<br>◯ Farmasys<br>◯ Sanday<br>◯ Iets anders
2.3.1	***If 'Welk apotheekinformatiesysteem (AIS) wordt er in uw apotheek gebruikt?' is equal to 'Iets anders' answer this question:***<br>U heeft bij de vorige vraag 'Iets anders' ingevuld. Licht dit toe.	

2.4	Hoeveel receptregels worden er gemiddeld per maand in uw apotheek verwerkt? U mag hier een schatting van geven. Deze vraag is niet verplicht.	◯ Minder dan 5000<br>◯ 5000 - 10.000<br>◯ 10.000 - 15.000<br>◯ 15.000 - 20.000<br>◯ 20.000 - 30.000<br>◯ 30.000 - 50.000<br>◯ Meer dan 50.000<br>◯ Zeg ik liever niet
2.5	Hoeveel FTE apotheker is op dit moment werkzaam in uw apotheek?	[ ] FTE
2.6	Welke functie heeft u?	◯ Apotheker<br>◯ Farmaceutisch consulent/manager<br>◯ Apotheekhoudende (huis)arts<br>◯ Apothekersassistent<br>◯ Apotheekmedewerker<br>◯ Iets anders
2.6.1	***If 'Welke functie heeft u?' is equal to 'Iets anders' answer this question:***<br>U heeft bij de vorige vraag 'Iets anders' ingevuld. Licht dit toe.	[ ]

Enquête medicatiebewaking en -overdracht van studiemedicatie - Openbare/poliklinische apotheek en apotheekhoudende huisarts - Inhoudelijke vragen

Number	Question	Answers
	Hierna volgen inhoudelijke vragen over medicatiebewaking en medicatieoverdracht van studiemedicatie.	
	Met studiemedicatie worden geneesmiddelen bedoeld, die in studieverband worden gebruikt. Het kan hierbij gaan om experimentele geneesmiddelen die nog niet geregistreerd zijn, of om bestaande geneesmiddelen die bijvoorbeeld voor een nieuwe indicatie worden onderzocht.	
	Met de openbare en poliklinische apotheek wordt er gedoeld op iedere apotheek waar patiënten laagdrempelig kunnen binnenlopen. In deze apotheek krijgt de patiënt ook de chronische medicatie geleverd en is het mogelijk om voorgeschreven medicatie van de huisarts en/of specialist op te halen.	
3.1	Hoe vaak per jaar krijgt u een melding van studiemedicatiegebruik van uw patiënten?	◯ Er wordt zover bekend geen studiemedicatie gebruikt door de patiënten van mijn apotheek.<br>◯ 1 - 2 keer per jaar<br>◯ 2 - 5 keer per jaar<br>◯ 5-10 keer per jaar<br>◯ Meer dan 10 keer per jaar

3.2	Staat in het kwaliteitshandboek iets beschreven over medicatiebewaking van studiemedicatie?	◯ Ja<br>◯ Nee
3.2.1	***If 'Staat in het kwaliteitshandboek iets beschreven over medicatiebewaking van studiemedicatie?' is equal to 'Ja' answer this question:***<br>Wat staat er in het kwaliteitshandboek beschreven over de medicatiebewaking van studiemedicatie?<br>Het kiezen van meerdere opties is mogelijk.	☐ Hoe de medicatiebewaking van studiemedicatie gedaan moet worden.<br>☐ Hoe studiemedicatiegebruik in het AIS gezet moet worden.<br>☐ Hoe studiemedicatiegebruik doorgegeven moet worden aan de huisarts.<br>☐ Iets anders.
3.2.1.1	***If 'Wat staat er in het kwaliteitshandboek beschreven over de medicatiebewaking van studiemedicatie?' is equal to 'Iets anders.' answer this question:***<br>U heeft bij de vorige vraag ‘Iets anders’ ingevuld. Licht dit toe.	
3.3	Op welke manier wordt u op de hoogte gesteld van het feit dat een patiënt meedoet aan een geneesmiddelstudie en gebruik maakt van studiemedicatie?<br>Het kiezen van meerdere opties is mogelijk.	☐ Via een mail/brief vanuit het ziekenhuis<br>☐ Mondeling vanuit het ziekenhuis<br>☐ Via een brief die u ontvangt van de patiënt<br>☐ Mondeling vanuit de patiënt<br>☐ Via de huisarts<br>☐ Via het LSP<br>☐ Ik word niet op de hoogte gesteld als een patiënt meedoet aan een geneesmiddelstudie en studiemedicatie gebruikt<br>☐ Dat is niet bekend<br>☐ Iets anders
3.3.1	***If 'Op welke manier wordt u op de hoogte gesteld van het feit dat een patiënt meedoet aan een geneesmiddelstudie en gebruik maakt van studiemedicatie?' is equal to 'Iets anders' answer this question:***<br>U heeft bij de vorige vraag ‘Iets anders’ ingevuld. Licht dit toe.	
3.4	Welke informatie heeft u ontvangen toen bericht kreeg over studiemedicatiegebruik door uw patiënt?	☐ Naam van het studiegeneesmiddel<br>☐ Dosering<br>☐ Mogelijke interacties<br>☐ Behandelduur<br>☐ Contactinformatie van de onderzoeker<br>☐ Ik heb nog nooit informatie gekregen over studiemedicatiegebruik door mijn patiënt<br>☐ Iets anders
3.3.2	***If 'Op welke manier wordt u op de hoogte gesteld van het feit dat een patiënt meedoet aan een geneesmiddelstudie en gebruik maakt van studiemedicatie?' is equal to 'Iets anders' answer this question:***<br>U heeft bij de vorige vraag ‘Iets anders’ ingevuld. Licht dit toe.	

3.4.1	***If 'Welke informatie heeft u ontvangen toen bericht kreeg over studiemedicatiegebruik door uw patiënt?' is not equal to 'Ik heb nog nooit informatie gekregen over studiemedicatiegebruik door mijn patiënt' answer this question:***<br>Miste u informatie om uw verantwoordelijkheden als apotheker te kunnen vervullen?	○ Ja<br>○ Nee
3.4.1.1	***If 'Miste u informatie om uw verantwoordelijkheden als apotheker te kunnen vervullen?' is equal to 'Ja' answer this question:***<br>U heeft bij de vorige vraag 'Ja' ingevuld. Ligt dit toe. Welke informatie miste u?	
3.5	Zijn er afspraken vastgelegd over het informeren van de apotheek door de huisarts over gebruik van studiemedicatie?	○ Ja<br>○ Nee
3.5.1	***If 'Zijn er afspraken vastgelegd over het informeren van de apotheek door de huisarts over gebruik van studiemedicatie?' is equal to 'Ja' answer this question:***<br>Ligt toe wat de afspraken met de huisarts zijn over de medicatiebewaking en -overdracht van studiemedicatie.	
3.6	Zijn er regionale afspraken gemaakt over de medicatiebewaking en -overdracht van studiemedicatie in uw regio?	○ Ja<br>○ Nee
3.6.1	***If 'Zijn er regionale afspraken gemaakt over de medicatiebewaking en -overdracht van studiemedicatie in uw regio?' is equal to 'Ja' answer this question:***<br>Licht toe wat de regionale afspraken zijn, die zijn gemaakt over de medicatiebewaking en -overdracht en van studiemedicatie?	
3.7	Indien u informatie heeft ontvangen over het studiemedicatiegebruk van een patiënt, wat gebeurt er met deze informatie?<br>Het kiezen van meerdere opties is mogelijk.	☐ Er wordt niks mee gedaan.<br>☐ Het studiemedicatiegebruik wordt ingevoerd in het AIS bij de patiënt als contra-indicatie.<br>☐ Het studiemedicatiegebruik wordt ingevoerd in het AIS bij de patiënt als memo of notitie.<br>☐ Het studiemedicatiegebruik wordt ingevoerd in het AIS bij de patiënt als ZZ-regel.<br>☐ De studiemedicatie wordt ingevoerd in het AIS bij de patiënt als actieve medicatie.<br>☐ Iets anders
3.7.1	***If 'Indien u informatie heeft ontvangen over het studiemedicatiegebruk van een patiënt, wat gebeurt er met deze informatie?' is equal to 'Iets anders' answer this question:***<br>U heeft bij de vorige vraag 'Iets anders' ingevuld. Licht dit toe.	

3.7.2	***If 'Indien u informatie heeft ontvangen over het studiemedicatiegebruk van een patiënt, wat gebeurt er met deze informatie?' is equal to 'Er wordt niks mee gedaan.' answer this question:***<br>Wat is de reden dat er geen actie wordt genomen als bekend wordt dat een patiënt studiemedicatie gebruikt?<br>Het kiezen van meerdere opties is mogelijk.	☐ Het is niet nuttig om deze informatie vast te leggen, want er wordt niet automatisch op bewaakt.<br>☐ Het is niet mogelijk om deze informatie in mijn AIS in te voeren.<br>☐ Er wordt geen vergoeding gegeven voor deze handeling.<br>☐ Ik weet niet wat ik zou moeten vastleggen.<br>☐ Studiemedicatiegebruik komt niet voor bij mij in de apotheek.<br>☐ Iets anders
3.7.2.1	***If 'Wat is de reden dat er geen actie wordt genomen als bekend wordt dat een patiënt studiemedicatie gebruikt?' is equal to 'Iets anders' answer this question:***<br>U heeft bij de vorige vraag 'Iets anders' ingevuld. Licht dit toe.	
3.8	Wat is het verschil in afhandeling bij geregistreerde en niet-geregistreerde studiemedicatie, indien aanwezig? Licht toe. Als er geen verschil is in afhandeling, mag er 'n.v.t.' ingevuld worden.<br><br>**Toelichting geregistreerde medicatie**<br><br>In Nederland geregistreerde geneesmiddelen zijn medicatie die een handelsvergunning hebben gekregen van de het College ter Beoordeling van Geneesmiddelen (CBG). Geneesmiddelen krijgen deze handelsvergunning pas wanneer deze geneesmiddelen zijn beoordeeld op basis van verschillende criteria, waaronder de effectiviteit. Deze geneesmiddelen zijn te vinden in de Geneesmiddeleninformatiebank op de website van de CBG en zijn te herkennen aan het registratienummer, wat begint met RVG.<br><br>Niet-geregistreerde geneesmiddelen hebben deze vergunning dus nog niet ontvangen. Dit kan bijvoorbeeld zijn wanneer een geneesmiddel nog wordt onderzocht in een klinische trial en dus experimenteel is, zoals het geval is bij studiemedicatie.	
3.9	Deze vraag betreft uw mening. Wat is er volgens u nodig om de medicatiebewaking van studiemedicatie en overdracht naar de eerste lijn goed uit te kunnen voeren?	
3.10	Deze vraag betreft uw mening. Welke rol zou de openbare/poliklinische apotheek volgens u moeten hebben in de medicatiebewaking en -overdracht van studiemedicatie?	

Enquête medicatiebewaking en -overdracht van studiemedicatie - Openbare/poliklinische apotheek en apotheekhoudende huisarts - Outro

Dit is het einde van de enquête.

Hartelijk dank voor uw medewerking aan deze enquête. Met uw hulp kunnen we de medicatiebewaking en -overdracht van studiemedicatie goed in kaart brengen. Hiermee kunnen we aanbevelingen doen voor een mogelijke landelijke richtlijn over studiemedicatiegebruik. Zo dragen we een steentje bij aan het verbeteren van veilig medicatiegebruik.

Bij verdere vragen kan er contact opgenomen met de uitvoerende onderzoeker via e-mail: a.j.elskamp@amsterdamumc.nl.

SUPPLEMENT 3: Empty eCRF – Hospital Pharmacy

Survey 'Enquête medicatiebewaking en -overdracht van studiemedicatie - Ziekenhuisapotheek'

Enquête medicatiebewaking en -overdracht van studiemedicatie - Ziekenhuisapotheek - Introduction

Welkom bij de enquête van het onderzoek 'Medicatiebewaking en medicatieoverdracht van studiemedicatie'. Het doel van dit onderzoek is het in kaart brengen van hoe de huidige medicatiebewaking en -overdracht van studiemedicatie op dit moment in Nederland is ingericht, met speciale aandacht voor hoe de ziekenhuisapotheek deze processen heeft ingericht. Dit is met het voornemen om aanbevelingen te kunnen doen voor een mogelijke nieuwe richtlijn omtrent studiemedicatie.

- Het invullen van de vragenlijst duurt ongeveer 5-10 minuten.
- De antwoorden worden gecodeerd verzameld en zijn niet herleidbaar naar u.
- Er wordt zorgvuldig omgegaan met de verzamelde gegevens, conform de [Gedragscode Gezondheidsonderzoek](https://www.coreon.org/wp-content/uploads/2022/01/Gedragscode-Gezondheidsonderzoek-2022.pdf).

Let op! Deze enquête hoeft maar *1 keer per medisch centrum* ingevuld te worden, mits het hele medische centrum dezelfde werkwijze hanteert betreft studiemedicatie. Mocht dit niet het geval zijn, dan graag *1 enquête per werkwijze* die aanwezig is in het medisch centrum de enquête invullen.

Dit onderzoek wordt uitgevoerd in opdracht van het Amsterdam UMC, afdeling Klinisch Geneesmiddelonderzoek. Bij vragen kan er contact opgenomen met de uitvoerende onderzoeker via e-mail: a.j.elskamp@amsterdamumc.nl.

Enquête medicatiebewaking en -overdracht van studiemedicatie - Ziekenhuisapotheek - Toestemming vragen

Number	Question	Answers
1.1	Hierbij geef ik toestemming voor het gebruik van de ingevulde gegevens voor onderzoek en ben ik me bewust dat de ingevulde gegevens gecodeerd worden verzameld en niet herleidbaar zijn naar mij of de organisatie waar ik werkzaam ben. Er zal zorgvuldig met de verzamelde gegevens opgegaan worden. Door 'Ik geef toestemming' te klikken, neemt u deel aan de enquête.	◯ Ik geef toestemming

Enquête medicatiebewaking en -overdracht van studiemedicatie - Ziekenhuisapotheek - Algemene vragen

Number	Question	Answers
2.1	In wat voor ziekenhuis bent u werkzaam?	◯ Academisch<br>◯ Topklinisch<br>◯ Perifeer
2.2	Hoeveel studies lopen er op dit moment in het ziekenhuis waar u werkzaam bent? U mag hier een schatting van geven.	

2.3	In welke provincie is het ziekenhuis waar u werkzaam in bent gevestigd?	◯ Drenthe<br>◯ Flevoland<br>◯ Friesland<br>◯ Gelderland<br>◯ Groningen<br>◯ Limburg<br>◯ Noord-Brabant<br>◯ Noord-Holland<br>◯ Overijssel<br>◯ Utrecht<br>◯ Zeeland<br>◯ Zuid-Holland
2.4	Welk ziekenhuisinformatiesysteem (HIS) wordt er gebruikt in het ziekenhuis waar u werkzaam bent?	◯ Epic (Beacon)<br>◯ HiX (ChipSoft)<br>◯ Nexus<br>◯ Cerner/SAP<br>◯ Iets anders
2.4.1	***If 'Welk ziekenhuisinformatiesysteem (HIS) wordt er gebruikt in het ziekenhuis waar u werkzaam bent?' is equal to 'Iets anders' answer this question:***<br>Als u bij de vorige vraag 'Iets anders' heeft ingevuld, licht dit toe.	

Enquête medicatiebewaking en -overdracht van studiemedicatie - Ziekenhuisapotheek - Medicatiebewaking

Number	Question	Answers
	**Hierna volgen inhoudelijke vragen over medicatiebewaking van studiemedicatie** De volgende vragen gaan over medicatiebewaking van studiemedicatie die tijdens de openingstijden van de ziekenhuisapotheek ter hand gesteld wordt. Decentrale uitgiftes of uitgiftes in de nacht vullen dus buiten de scope van dit onderzoek.	
3.1	Is de ziekenhuisapotheek betrokken bij het uitvoeren van de interactiecontrole van studiemedicatie?	◯ Ja<br>◯ Nee
3.1.1	***If 'Is de ziekenhuisapotheek betrokken bij het uitvoeren van de interactiecontrole van studiemedicatie?' is equal to 'Ja' answer this question:***<br>Wanneer is de ziekenhuisapotheek betrokken bij het uitvoeren van interactiecontrole van studiemedicatie?	☐ Bij de eerste uitgifte (EU) van studiemedicatie<br>☐ Bij de tweede uitgifte (TU) van studiemedicatie<br>☐ Bij iedere vervolguitgifte (VU) van studiemedicatie<br>☐ Bij de EU van thuismedicatie naast gebruik van studiemedicatie

3.1.2	***If 'Is de ziekenhuisapotheek betrokken bij het uitvoeren van de interactiecontrole van studiemedicatie?' is equal to 'Nee' answer this question:***<br>Waarom is de ziekenhuisapotheek niet betrokken bij het uitvoeren van de interactiecontrole van studiemedicatie?	☐ De interactiecontrole van studiemedicatie wordt door de onderzoeker gedaan.<br>☐ De interactiecontrole is al gewaarborgd in het protocol van het onderzoek.<br>☐ Het is niet mogelijk om de interactiecontrole van studiemedicatie te doen, omdat de studiemedicatie niet in het ZIS ingevoerd kan worden.<br>☐ Het is niet mogelijk om de interactiecontrole van studiemedicatie te doen, omdat er geen actueel medicatieoverzicht van de patiënt beschikbaar is.<br>☐ Iets anders.
3.1.3	***If 'Is de ziekenhuisapotheek betrokken bij het uitvoeren van de interactiecontrole van studiemedicatie?' is equal to 'Nee' answer this question:***<br>U heeft bij de vorige vraag ‘Iets anders’ ingevuld. Licht dit toe.	
3.1.1.1	***If 'Wanneer is de ziekenhuisapotheek betrokken bij het uitvoeren van interactiecontrole van studiemedicatie?' is equal to 'Bij de eerste uitgifte (EU) van studiemedicatie' answer this question:***<br>Hoe wordt de interactiecontrole gedaan bij de EU van studiemedicatie?	☐ Door middel van het studieprotocol.<br>☐ Door een (online) interactiechecker te gebruiken<br>☐ Door het in te voeren in het ZIS (dus aan de hand van de interactielijsten in het ZIS).<br>☐ Aan de hand van vooraf opgestelde instructie/protocollen.<br>☐ Iets anders.
3.1.1.1.1	***If 'Hoe wordt de interactiecontrole gedaan bij de EU van studiemedicatie?' is equal to 'Iets anders.' answer this question:***<br>U heeft bij de vorige vraag ‘Iets anders’ ingevuld. Licht dit toe.	
3.1.1.2	***If 'Wanneer is de ziekenhuisapotheek betrokken bij het uitvoeren van interactiecontrole van studiemedicatie?' is equal to 'Bij de eerste uitgifte (EU) van studiemedicatie' answer this question:***<br>Welk verschil zit er in de interactiecontrole van wel geregistreerde en niet-geregistreerde studiemedicatie bij de EU?	☐ Er wordt **wel** gecontroleerd bij geregistreerde studiemedicatie, maar er wordt **niet** gecontroleerd bij niet-geregistreerde studiemedicatie.<br>☐ Er wordt **wel** gecontroleerd bij niet-geregistreerde studiemedicatie, maar er wordt **niet** gecontroleerd bij geregistreerde studiemedicatie.<br>☐ De interactiecontrole van niet-geregistreerde studiemedicatie wordt gedaan a.d.h.v. het studieprotocol, terwijl de interactiecontrole van geregistreerde studiemedicatie via de interactielijsten van het ZIS wordt gedaan.<br>☐ Er zit **geen** verschil tussen de interactiecontrole geregistreerde en niet-geregistreerde studiemedicatie.<br>☐ Iets anders.

3.1.1.2.1	***If 'Welk verschil zit er in de interactiecontrole van wel geregistreerde en niet-geregistreerde studiemedicatie bij de EU?' is equal to 'Iets anders.' answer this question:*** U heeft bij de vorige vraag 'Iets anders' ingevuld. Licht dit toe.	
3.1.1.3	***If 'Wanneer is de ziekenhuisapotheek betrokken bij het uitvoeren van interactiecontrole van studiemedicatie?' is equal to 'Bij de eerste uitgifte (EU) van studiemedicatie' answer this question:*** Door wie wordt de interactiecontrole bij de EU van studiemedicatie uitgevoerd?	☐ Een (KGO-)apotheker<br>☐ De onderzoeker<br>☐ Dat weet ik niet<br>☐ Iets anders.
3.1.1.3.1	***If 'Door wie wordt de interactiecontrole bij de EU van studiemedicatie uitgevoerd?' is equal to 'Iets anders.' answer this question:*** U heeft bij de vorige vraag 'Iets anders' ingevuld. Licht dit toe.	
3.1.1.4	***If 'Wanneer is de ziekenhuisapotheek betrokken bij het uitvoeren van interactiecontrole van studiemedicatie?' is equal to 'Bij de tweede uitgifte (TU) van studiemedicatie' answer this question:*** Hoe wordt de interactiecontrole gedaan bij de TU van studiemedicatie?	☐ Door middel van het studieprotocol.<br>☐ Door een (online) interactiechecker te gebruiken<br>☐ Door het in te voeren in het ZIS (dus aan de hand van de interactielijsten in het ZIS).<br>☐ Aan de hand van vooraf opgestelde instructie/protocollen.<br>☐ Iets anders.
3.1.1.4.1	***If 'Hoe wordt de interactiecontrole gedaan bij de TU van studiemedicatie?' is equal to 'Iets anders.' answer this question:*** U heeft bij de vorige vraag 'Iets anders' ingevuld. Licht dit toe.	
3.1.1.5	***If 'Wanneer is de ziekenhuisapotheek betrokken bij het uitvoeren van interactiecontrole van studiemedicatie?' is equal to 'Bij de tweede uitgifte (TU) van studiemedicatie' answer this question:*** Welk verschil zit er in de interactiecontrole van wel geregistreerde en niet-geregistreerde studiemedicatie bij de TU?	☐ Er wordt **wel** gecontroleerd bij geregistreerde studiemedicatie, maar er wordt **niet** gecontroleerd bij niet-geregistreerde studiemedicatie.<br>☐ Er wordt **wel** gecontroleerd bij niet-geregistreerde studiemedicatie, maar er wordt **niet** gecontroleerd bij geregistreerde studiemedicatie.<br>☐ De interactiecontrole van niet-geregistreerde studiemedicatie wordt gedaan a.d.h.v. het studieprotocol, terwijl de interactiecontrole van geregistreerde studiemedicatie via de interactielijsten van het ZIS wordt gedaan.<br>☐ Er zit **geen** verschil tussen de interactiecontrole geregistreerde en niet-geregistreerde studiemedicatie.<br>☐ Iets anders.

3.1.1.5.1	***If 'Welk verschil zit er in de interactiecontrole van wel geregistreerde en niet-geregistreerde studiemedicatie bij de TU?' is equal to 'Iets anders.' answer this question:*** U heeft bij de vorige vraag 'Iets anders' ingevuld. Licht dit toe.	______________
3.1.1.6	***If 'Wanneer is de ziekenhuisapotheek betrokken bij het uitvoeren van interactiecontrole van studiemedicatie?' is equal to 'Bij de tweede uitgifte (TU) van studiemedicatie' answer this question:*** Door wie wordt de interactiecontrole bij de TU van studiemedicatie uitgevoerd?	☐ Een (KGO-)apotheker<br>☐ De onderzoeker<br>☐ Dat weet ik niet<br>☐ Iets anders.
3.1.1.6.1	***If 'Door wie wordt de interactiecontrole bij de TU van studiemedicatie uitgevoerd?' is equal to 'Iets anders.' answer this question:*** U heeft bij de vorige vraag 'Iets anders' ingevuld. Licht dit toe.	______________
3.1.1.7	***If 'Wanneer is de ziekenhuisapotheek betrokken bij het uitvoeren van interactiecontrole van studiemedicatie?' is equal to 'Bij iedere vervolguitgifte (VU) van studiemedicatie' answer this question:*** Hoe wordt de interactiecontrole gedaan bij een VU van studiemedicatie?	☐ Door middel van het studieprotocol.<br>☐ Door een (online) interactiechecker te gebruiken<br>☐ Door het in te voeren in het ZIS (dus aan de hand van de interactielijsten in het ZIS).<br>☐ Aan de hand van vooraf opgestelde instructie/protocollen.<br>☐ Iets anders.
3.1.1.7.1	***If 'Hoe wordt de interactiecontrole gedaan bij een VU van studiemedicatie?' is equal to 'Iets anders.' answer this question:*** U heeft bij de vorige vraag 'Iets anders' ingevuld. Licht dit toe.	______________
3.1.1.8	***If 'Wanneer is de ziekenhuisapotheek betrokken bij het uitvoeren van interactiecontrole van studiemedicatie?' is equal to 'Bij iedere vervolguitgifte (VU) van studiemedicatie' answer this question:*** Welk verschil zit er in de interactiecontrole van wel geregistreerde en niet-geregistreerde studiemedicatie bij een VU?	☐ Er wordt **wel** gecontroleerd bij geregistreerde studiemedicatie, maar er wordt **niet** gecontroleerd bij niet-geregistreerde studiemedicatie.<br>☐ Er wordt **wel** gecontroleerd bij niet-geregistreerde studiemedicatie, maar er wordt **niet** gecontroleerd bij geregistreerde studiemedicatie.<br>☐ De interactiecontrole van niet-geregistreerde studiemedicatie wordt gedaan a.d.h.v. het studieprotocol, terwijl de interactiecontrole van geregistreerde studiemedicatie via de interactielijsten van het ZIS wordt gedaan.<br>☐ Er zit **geen** verschil tussen de interactiecontrole geregistreerde en niet-geregistreerde studiemedicatie.<br>☐ Iets anders.

3.1.1.8.1	***If 'Welk verschil zit er in de interactiecontrole van wel geregistreerde en niet-geregistreerde studiemedicatie bij een VU?' is equal to 'Iets anders.' answer this question:***<br>U heeft bij de vorige vraag 'Iets anders' ingevuld. Licht dit toe.	
3.1.1.9	***If 'Wanneer is de ziekenhuisapotheek betrokken bij het uitvoeren van interactiecontrole van studiemedicatie?' is equal to 'Bij iedere vervolguitgifte (VU) van studiemedicatie' answer this question:***<br>Door wie wordt de interactiecontrole bij iedere VU van studiemedicatie uitgevoerd?	☐ Een (KGO-)apotheker<br>☐ De onderzoeker<br>☐ Dat weet ik niet<br>☐ Iets anders.
3.1.1.9.1	***If 'Door wie wordt de interactiecontrole bij iedere VU van studiemedicatie uitgevoerd?' is equal to 'Iets anders.' answer this question:***<br>U heeft bij de vorige vraag 'Iets anders' ingevuld. Licht dit toe.	
3.1.1.10	***If 'Wanneer is de ziekenhuisapotheek betrokken bij het uitvoeren van interactiecontrole van studiemedicatie?' is equal to 'Bij de EU van thuismedicatie naast gebruik van studiemedicatie' answer this question:***<br>Hoe doet de ziekenhuisapotheek de in interactiecontrole van thuismedicatie, die is voorgeschreven tijdens het gebruik van studiemedicatie?	☐ Door middel van het studieprotocol.<br>☐ Door een (online) interactiechecker te gebruiken<br>☐ Door het in te voeren in het ZIS (dus aan de hand van de interactielijsten in het ZIS).<br>☐ Aan de hand van vooraf opgestelde instructie/protocollen.<br>☐ Iets anders.
3.1.1.10.1	***If 'Hoe doet de ziekenhuisapotheek de in interactiecontrole van thuismedicatie, die is voorgeschreven tijdens het gebruik van studiemedicatie?' is equal to 'Iets anders.' answer this question:***<br>U heeft bij de vorige vraag 'Iets anders' ingevuld. Licht dit toe.	
3.1.1.11	***If 'Wanneer is de ziekenhuisapotheek betrokken bij het uitvoeren van interactiecontrole van studiemedicatie?' is equal to 'Bij de EU van thuismedicatie naast gebruik van studiemedicatie' answer this question:***<br>Door wie wordt de interactiecontrole bij een EU van thuismedicatie naast studiemedicatiegebruik uitgoeverd?	☐ Een (KGO-)apotheker<br>☐ De onderzoeker<br>☐ Dat weet ik niet<br>☐ Iets anders.

3.1.1.11.1	***If 'Door wie wordt de interactiecontrole bij een EU van thuismedicatie naast studiemedicatiegebruik uitgoeverd?' is equal to 'Iets anders.' answer this question:***<br>U heeft bij de vorige vraag ‘Iets anders’ ingevuld. Licht dit toe.	
3.2	Deze vraag betreft uw mening. Vindt u dat **de apotheker** verantwoordelijk is voor de medicatiebewaking van studiemedicatie?	1 (=helemaal oneens) (1) — 5 (=helemaal mee eens) (5)
3.3	Deze vraag betreft uw mening. Vindt u dat **de onderzoeker** verantwoordelijk is voor de medicatiebewaking van studiemedicatie?	1 (=helemaal oneens) (1) — 5 (=helemaal mee eens) (5)
3.4	Deze vraag betreft uw mening. Op welke manier zou het proces van medicatiebewaking van studiemedicatie volgens u verbeterd kunnen worden?	

Enquête medicatiebewaking en -overdracht van studiemedicatie - Ziekenhuisapotheek - Medicatieoverdracht

Number	Question	Answers
	**Nu volgen er inhoudelijke vragen over medicatieoverdracht van studiemedicatie.** Met de thuisapotheek wordt iedere apotheek bedoeld waar patiënten laagdrempelig kunnen binnenlopen. In de thuisapotheek krijgt de patiënt ook de chronische medicatie geleverd en is het mogelijk om voorgeschreven medicatie van de huisarts en/of specialist op te halen. Dit gaat dus om openbare apotheken, poliklinische apotheken en apotheekhoudende huisartsen.	
4.1	Wordt er informatie **door de ziekenhuisapotheek** overgedragen naar de thuisapotheek over studiemedicatiegebruik?	○ Ja<br>○ Nee
4.1.1	***If 'Wordt er informatie door de ziekenhuisapotheek overgedragen naar de thuisapotheek over studiemedicatiegebruik?' is equal to 'Ja' answer this question:***<br>Op welke manier wordt het studiemedicatiegebruik overgedragen naar de thuisapotheek?	☐ De ziekenhuisapotheek stuurt een bericht naar de thuisapotheek.<br>☐ De ziekenhuisapotheek stuurt een bericht naar de huisarts.<br>☐ De ziekenhuisapotheek geeft een brief mee aan de patiënt om in te leveren bij de thuisapotheek.<br>☐ De ziekenhuisapotheek geeft het door via het LSP.<br>☐ De medicatieoverdracht gaat via de poliklinische apotheek.<br>☐ De patiënt wordt verteld om dit door te geven aan de eerste lijn.<br>☐ Iets anders

4.1.1.1	***If 'Op welke manier wordt het studiemedicatiegebruik overgedragen naar de thuisapotheek?' is equal to 'Iets anders' answer this question:***<br>U heeft bij de vorige vraag 'Iets anders' ingevuld. Licht dit toe.	
4.1.2	***If 'Wordt er informatie door de ziekenhuisapotheek overgedragen naar de thuisapotheek over studiemedicatiegebruik?' is equal to 'Nee' answer this question:***<br>Waarom wordt er vanuit de ziekenhuisapotheek geen informatie overgedragen over studiemedicatiegebruik naar de thuisapotheek?	☐ De medicatieoverdracht wordt gedaan door de onderzoeker.<br>☐ De medicatieoverdracht wordt gedaan door de poliklinische apotheek.<br>☐ De medicatieoverdracht wordt geregeld door de patiënt zelf.<br>☐ Het is niet nodig om de thuisapotheek te informeren over studiemedicatiegebruik.<br>☐ Iets anders
4.1.2.1	***If 'Waarom wordt er vanuit de ziekenhuisapotheek geen informatie overgedragen over studiemedicatiegebruik naar de thuisapotheek?' is equal to 'Iets anders' answer this question:***<br>U heeft bij de vorige vraag 'Iets anders' ingevuld. Licht dit toe.	
4.2	Deze vraag betreft uw mening. Vindt u **de apotheker** verantwoordelijk voor de medicatieoverdracht van studiemedicatie naar de thuisapotheek?	1(= geheel oneens) (1) ——— 5(= geheel mee eens) (5)
4.3	Deze vraag betreft uw mening. Vindt u **de onderzoeker** verantwoordelijk voor de medicatieoverdracht van studiemedicatie naar de thuisapotheek?	1(= geheel oneens) (1) ——— 5(= geheel mee eens) (5)
4.4	Zijn er regionale afspraken met de eerste lijn omtrent medicatieoverdracht van studiemedicatie?	○ Ja<br>○ Nee
4.4.1	***If 'Zijn er regionale afspraken met de eerste lijn omtrent medicatieoverdracht van studiemedicatie?' is equal to 'Ja' answer this question:***<br>Welke regionale afspraken zijn gemaakt omtrent studiemedicatie?	
4.5	Deze vraag betreft uw mening. Op welke manier zou het proces van medicatieoverdracht van studiemedicatie volgens u verbeterd kunnen worden? Of antwoord 'niet van toepassing' indien er volgens u geen aanpassing in huidige proces nodig zijn.	

Enquête medicatiebewaking en -overdracht van studiemedicatie - Ziekenhuisapotheek - Outro

Dit is het einde van de enquête.

Hartelijk dank voor uw medewerking aan deze enquête. Met uw hulp kunnen we de medicatiebewaking en -overdracht van studiemedicatie goed in kaart brengen. Hiermee kunnen we aanbevelingen doen voor een mogelijke landelijke richtlijn over studiemedicatiegebruik. Hiermee dragen we een steentje bij aan het verbeteren van veilig medicatiegebruik.

Bij verdere vragen kan er contact opgenomen met de uitvoerende onderzoeker via e-mail: a.j.elskamp@amsterdamumc.nl.

SUPPLEMENT 4: Interview scheme

F1.3 Interviewschema Onderzoekers
Versie 2 dd 17-12-2024
Introductie

- Voorstellen
  - Naam
  - Research stagiair: onderzoek doen voor een half jaar
  - Onder begeleiding van Niels en Anne-Jaël van de apotheek, afdeling Klinisch GeneesmiddelOnderzoek (onderzoekscentrum VUmc).
- Het gesprek zal 15-20 minuten duren.
- De informatie die in dit gesprek besproken wordt, zal wel gebruikt worden in het onderzoek, maar de gegevens zullen niet terug te leiden zijn naar u als persoon.
  - Ook beter als de onderzoeker zelf niet zijn/haar naam noemt tijdens het opnemen va het gesprek.
- Vindt u het goed als ik dit gesprek opneem?

(i.v.m. het later verwerken van de gegevens).

Vragen (introducerend)

- Met wat voor onderzoek houdt u zich nu bezig? Welke soort geneesmiddelen worden er onderzocht in uw onderzoek?
  - Wel/niet geregistreerd (welke fase studie)
  - Hoog/laag risico (welke fase)
- Voor hoeveel onderzoeken bent u op dit moment verantwoordelijk?
- Wat is de achtergrond van de onderzoeker? Arts? Biomedisch wetenschapper? Apotheker?

Vragen (proces)
Medicatiebewaking

- Hoe wordt op dit moment de medicatiebewaking van uw onderzoeksparticipanten in uw onderzoek uitgevoerd?
  - Welk moment?
- ▪ Bij inclusie
- ▪ Eerste uitgifte en vervolguitgifte
- ▪ Gelijktijdig gebruik van thuismedicatie (stel ze krijgen wat nieuws voorgeschreven vanuit de eerste lijn)
  - Door wie (betrokkenheid apotheker)
- ▪ Is er een apotheker betrokken bij de medicatiebewaking?
- ▪ Hoe komt u aan de benodigde expertise die nodig is om de medicatiebewaking uit te kunnen voeren?
- ▪ Wat verwacht u van de ziekenhuisapotheek? (degene die het aflevert).
- Studiespecifieke afspraken:
  - Wat voor instructies krijgt de onderzoeksdeelnemer naar huis betreft het actueel medicatieoverzicht?
  - Stel de onderzoeksdeelnemer wordt ziek, welke instructies krijgen zij?
- ▪ Moeten ze dat direct melden bij de onderzoeker?
- ▪ Mogen ze naar de huisarts?

Medicatieoverdracht

- Welke acties voeren jullie op dit moment uit om studiemedicatiegebruik van de onderzoeksdeelnemers door te geven aan de eerste lijn? (Huisarts, thuisapotheek, etc.)
  - Instructies aan de patiënt zelf?

    - In het LSP of AIS gezet?
- Wat verwacht u dat de thuisapotheek doet met deze informatie?
- Wat zou u willen dat de thuisapotheek doet met deze informatie?
- Zijn er regionale afspraken wat betreft medicatieoverdracht van studiemedicatie?

Vragen (mening)

- Bent u tevreden met hoe de medicatiebewaking en overdracht van studiemedicatie op dit moment gaat?
- Tegen welke uitdagingen loopt u aan bij het uitvoeren van de medicatiebewaking en -overdracht van uw onderzoeksdeelnemers?
- Wat zou u nodig hebben om het uitvoeren van de medicatiebewaking en -overdracht van uw onderzoeksdeelnemers uit te kunnen voeren?
- Wie zou volgens u verantwoordelijk moeten zijn voor de medicatiebewaking en -overdracht van studiemedicatie?
    - Onderzoeker vs. thuis- en ziekenhuisapotheek
    - Gedeelde verantwoordelijkheid? Verdeling van taken?
    - Samenwerken? Apotheker in onderzoeksteam?

Afsluiting

- Bedanken voor de tijd
- Informatie geven voor hoe en wat verder
    - Wilt u op de hoogte blijven van dit onderzoek? Mogen we dan uw contactgegevens opslaan?
    - Wanneer het artikel wordt verwacht.
- Het interview wordt anoniem uitgevoerd. Het is niet nodig of de bedoeling dat de geïnterviewde hun naam deelt.
